# Investigation of the inflammatory cell migration process in familial Mediterranean fever

**DOI:** 10.1186/1546-0096-13-S1-O13

**Published:** 2015-09-28

**Authors:** YZ Akkaya Ulum, E Avci, ED Batu, O Karadag, S Ozen, N Purali, E Yilmaz, B Balci Peynircioglu

**Affiliations:** 1 Hacettepe University, Medical Biology, Ankara, Turkey; 2 Hacettepe University, Pediatric Rheumatology, Ankara, Turkey; 3 Hacettepe University, Rheumatology, Ankara, Turkey; 4 Hacettepe University, Biophysics, Ankara, Turkey

## Introduction

Familial Mediterranean fever (FMF) is one of the most common autoinflammatory disorders and is characterized by episodic attacks of fever, along with inflammation. FMF pathogenesis is associated with various mutations in the MEFV gene, which encodes pyrin. Pyrin is expressed predominantly in neutrophils that have an important role in the innate immune response. Several proteins related with actin machinery have been identified as pyrin-interacting proteins in our *in vitro* cell migration models. Thus, in this study, we hypothesized that pyrin may have a key role in neutrophil migration during inflammation and decided to do functional analysis on pyrin silenced cell lines and primary neutrophils.

## Objectives

In this study, we aimed to analyze the possible role of Pyrin in cell migration process in both neutrophil cell line and primary neutrophil cells isolated from FMF patients.

## Patients and methods

HL-60 cells, a neutrophil-like cell line, were cultured and differentiated. MEFV gene was silenced with MEFV siRNA. The expression level of pyrin was assessed by using western blot. Cells were stimulated for migration using fMLP (N-formyl-Met-Leu-Phe) after that cells were co-stained with pyrin and actin to see polarization. Colocalizations were analyzed by drawing profile and correlation curves with the help of confocal microscopy. Blood samples were collected from 2 controls and 3 M694V/M694V patients. Neutrophil cells were isolated with Lympholyte-poly solution. A modified Boyden Chamber assay was used to detect chemotaxis range of the neutrophils. Equal numbers of cells were migrated towards the gradient of fMLP for 24 hours. Then the migrated cells were stained by 4 uM calcein-AM and visualized under fluorescence microscopy.

## Results

According to the MEFV siRNA experiments, pyrin was silenced in HL-60 cells with %80 efficiency. In this high efficiency rate, when cells stimulated for migration, they showed less actin polymerization compared to control cells and appeared as round shape instead of having polarized shape. Chemotaxis experiments using primary neutrophils showed that the migration rates of patients’ neutrophil cells were 6 times higher than the controls (p<0.05). The average of the migrated cell numbers in patient and control group was 38 x 10^3^ and 5 x 10^3^, respectively.

## Conclusion

We have demonstrated that mutant pyrin causes an increase in the neutrophil migration rate. Besides, when pyrin is silenced, the ability of the cell migration is decreased and the cells get less polarized shape. Thus these results suggest a pro-inflammatory role of pyrin in the regulation of inflammation by influencing the cell migration process possibly at the early phase of the migration by interaction with actin. In conclusion, the studies described here provide a new insight to the potential role of pyrin protein in the process of neutrophil migration during inflammation.

